# The invasion, provenance and diversity of *Vespa velutina* Lepeletier (Hymenoptera: Vespidae) in Great Britain

**DOI:** 10.1371/journal.pone.0185172

**Published:** 2017-09-26

**Authors:** Giles E. Budge, Jennifer Hodgetts, Eleanor P. Jones, Jozef C. Ostojá-Starzewski, Jayne Hall, Victoria Tomkies, Nigel Semmence, Mike Brown, Maureen Wakefield, Kirsty Stainton

**Affiliations:** 1 Fera, The National Agrifood Innovation Campus, Sand Hutton, York, United Kingdom; 2 Institute for Agri-Food Research and Innovation, Newcastle University, Newcastle upon Tyne, United Kingdom; 3 National Bee Unit, Animal and Plant Health Agency, The National Agrifood Innovation Campus, Sand Hutton, York, United Kingdom; University of Cologne, GERMANY

## Abstract

The yellow-legged or Asian hornet (*Vespa velutina* colour form *nigrithorax*) was introduced into France from China over a decade ago. *Vespa velutina* has since spread rapidly across Europe, facilitated by suitable climatic conditions and the ability of a single nest to disperse many mated queens over a large area. Yellow-legged hornets are a major concern because of the potential impact they have on populations of many beneficial pollinators, most notably the western honey bee (*Apis mellifera*), which shows no effective defensive behaviours against this exotic predator. Here, we present the first report of this species in Great Britain. Actively foraging hornets were detected at two locations, the first around a single nest in Gloucestershire, and the second a single hornet trapped 54 km away in Somerset. The foraging activity observed in Gloucestershire was largely restricted to within 700 m of a single nest, suggesting highly localised movements. Genetic analyses of individuals from the Gloucestershire nest and the single hornet from Somerset suggest that these incursions represent an expansion of the European population, rather than a second incursion from Asia. The founding queen of the Gloucestershire nest mated with a single male, suggesting that sexual reproduction may have occurred in an area of low nest density. Whilst the nest contained diploid adult males, haploid ‘true’ males were only present at the egg stage, indicating that the nest was detected and removed before the production of queens. Members of the public reported additional dead hornets associated with camping equipment recently returned from France and imported timber products, highlighting possible pathways of incursion. The utility of microsatellites to inform surveillance during an incursion and the challenge of achieving eradication of this damaging pest are discussed.

## Introduction

Many Vespidae predators have become damaging invasive species capable of causing negative ecological, economic and social impacts [1]. Eusocial insects represent only about 2% of global insect species, yet over 24% the alien insect species causing environmental effects are eusocial [1]. The yellow-legged or Asian hornet (*V*. *velutina*) is one such species that has expanded from its native range in South East Asia [2] to invade multiple new territories including South Korea [3] and Japan [4].

*V*. *velutina* colour form *nigrithorax* Du Buysson is the first Vespidae predator accidentally introduced from Asia into Europe [1]. The actual route of incursion into Europe can never be truly known, but is thought to have occurred in 2004 in south-west France, after accidental importation in Bonsai pots from China [5, 6]. This species has since spread rapidly across Europe with reports from Spain in 2010 [7], Belgium and Portugal in 2011 [8, 9], Italy in 2013 [10], Germany in 2014 [11] and Switzerland in 2017 [12]. *Vespa velutina* is a predator of beneficial insects including bumblebees (*Bombus* spp.), sweat bees (Halticidae), wasps, brachycera flies, and particularly the western honey bee (*Apis mellifera* L.) [13]. Hornet predation can weaken and kill honey bee colonies, although these impacts are difficult to quantify [14].

*V*. *velutina* nests are founded by a single mated queen. At the end of the summer, each successful nest produces multiple queens, which are mated and hibernate over winter. Queens hibernate in crevices, tree bark and soil [15, 16], and therefore many commodities are capable of inadvertently moving queens, which gives rise to many possible routes of entry for invasion into new territories [17]. Whilst multiple nests of *V*. *velutina* have been known to establish within 1 km of apiaries containing foraging hornets, it has not been possible to determine the foraging range from a single nest when this pest is well established [18].

Here we report the first incursions of *V*. *velutina* in Great Britain and use a combination of field observations and genetic analyses to highlight foraging habits, likely origin, nest diversity and observed incursion pathways.

## Methods

### Observations of foraging hornets

On 17^th^ September 2016, a suspected yellow-legged hornet was reported foraging near an apiary by a beekeeper in Tetbury, Gloucestershire UK (sample name ‘*Tetbury1’*). Government inspectors from the National Bee Unit (NBU) instigated local surveillance at potential forage sites and confirmed multiple sightings of foraging hornets, suggesting an active nest. Land owner permissions were obtained where field observations required access to private land. The nest was found on 28^th^ September in a cypress tree, treated using Ficam D (Bendiocarb), and removed on 30^th^ September.

On 29^th^ of September, a beekeeper in Somerset (a distance of 54 km from the Tetbury nest) reported they had trapped a single foraging hornet between April and June 2016 (sample name ‘*Somerset’*).

### Dead hornet samples

**Fig 1:**
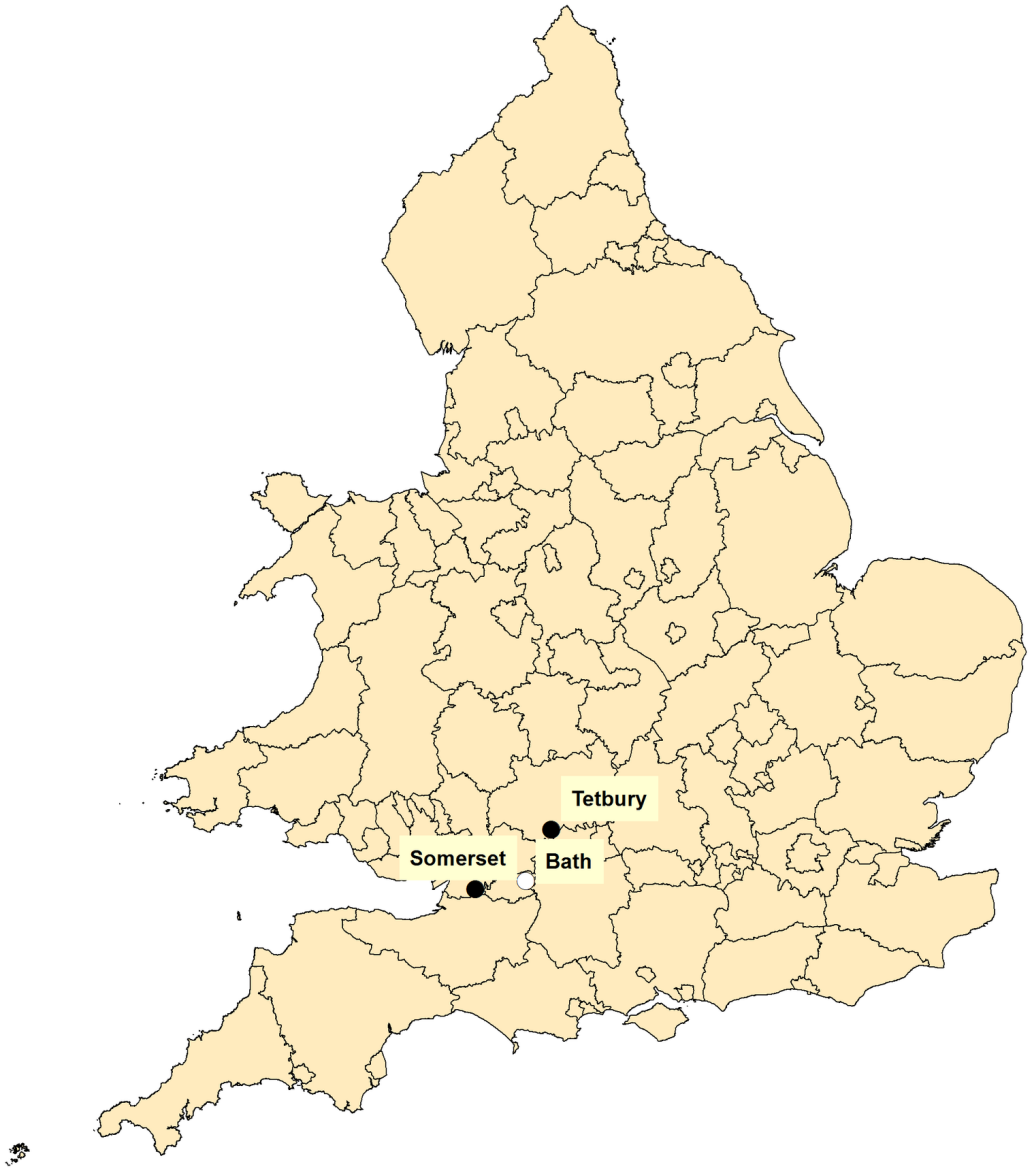
*Vespa velutina* locations. *Vespa velutina* locations for the active nest in Tetbury, Gloucester (black circle), the single trapped foraging hornet in Somerset (black circle), and the dead hornet found in Bath after being inadvertently imported in camping equipment (white circle).

On 7^th^ October, a dead yellow-legged hornet was found near Tetbury in a stack of wood recently imported from the Loire Valley, France (sample name *‘Tetbury2’*). The wood had been treated with Boron before importation and had been onsite for several weeks. On 13^th^ October, a dead yellow-legged hornet was found in some camping equipment near Bath (sample name ‘*Bath*’) that had returned from the Loire valley a week earlier. No radial searches were deployed. Hornet locations are shown in Fig 1.

Each hornet was identified using morphological keys [2, 19] and comparison to reference specimens from France (courtesy of Quentin Rome, Muséum National d'Histoire Naturelle, Paris).

### Nest observations, sampling and DNA extraction

The nest from Tetbury was examined and the number of adult hornets, gender ratio, weight and nest size recorded. Individual adult hornets were weighed using an analytical balance (Sartorius R2000; ± 0.1 mg). In total, 19 adult females and seven adult males were weighed as found in the nest and therefore some air drying of individuals may have occurred after death. Also six additional individual females were dried in an oven until there was no further change in mass to obtain the dry weight. Adults, teneral adults, pupae, larvae and eggs were sampled and DNA extracted using the DNeasy^®^ blood and tissue kit (QIAGEN) following the manufacturer’s protocol for the extraction of total DNA from animal tissues using spin columns. Tissue was first homogenised with a micro-pestle prior to overnight lysis and DNA was eluted in 200 μl of buffer AE.

### Cytochrome Oxidase I sequencing

PCR of the established invertebrate DNA barcode region of the Cytochrome C Oxidase Subunit I (COI) gene was undertaken using two independent primer pairs: LCO1490/HCO2198 [20] and LepF/LepR [21]. All PCR reactions (25 μl) were performed in a GeneAmp^®^ 9700 thermocycler (Applied Biosystems) using a proof-reading DNA polymerase, and comprised: 12.5 μl 2x BIO-X-ACT short PCR mix (Bioline), 400 nm of each primer and 1 μl DNA (concentration as extracted). All primers were synthesised by Eurofins-MWG-Operon. PCR cycling conditions were 5 min at 94°C; followed by 35 cycles of 30 sec at 94°C, 45 sec at 50°C, and 1 min at 72°C; and a final elongation step of 10 min at 72°C. PCR products (5 μl) were separated by agarose gel electrophoresis, and the remainder purified using the QIAquick^®^ PCR purification kit (QIAGEN) prior to sequencing both strands using the PCR primers by Eurofins-MWG-Operon. DNA sequence analysis and alignments were performed using Geneious^®^ 9.1.4 [22] and a consensus sequence created. Each nucleotide position had four times coverage (forward and reverse from two primer sets) and was assessed for sequence similarity against GenBank^®^ using the Basic Local Alignment Search Tool (BLAST; blastn algorithm; http://www.ncbi.nlm.nih.gov/blast) and the BOLD database (http://www.boldsystems.org/). All nucleotide sequences were submitted to GenBank.

A reference alignment of 20 *V*. *velutina* sequences from GenBank and sequences from the four UK interceptions was created using the Geneious alignment tool within Geneious^®^ 9.1.4 [22] and trimmed to 636 nucleotides. A Maximum Likelihood (ML) phylogenetic tree was generated in PhyML with 1000 bootstrap replicates [23], using the PhyML SMS tool to select the model of nucleotide substitution. The resulting tree was rooted with *V*. *bicolor* (accession KT257112), *V*. *vivax* (accession KT257116) and *V*. *affinis* (accession KJ147242).

### Microsatellite marker analysis

Samples were genotyped for 15 microsatellite markers: R1-36, LIST2020, R1-80, R4-33, R4-114, R1-169, D2-185, LIST2018, D3-15, VMA6, LIST2015, R4-26, R1-75, R4-100, VMA8 [24]. Microsatellites were tagged with FAM, NED or HEX fluorescent dyes and were run in six multiplexed PCR reactions using the Qiagen Multiplex PCR kits (Table 1). Primer concentrations were optimised for each multiplex; cycling conditions were 15 min at 95°C, followed by 35 cycles of 30 sec at 94°C, 90 sec at 59°C, 60 sec at 72°C, and a final elongation step of 30 min at 60°C. PCR products were genotyped on an ABI 3031 Genetic Analyzer using the ROX 500 ladder as a size standard. Genotypes were scored into allele sizes using Geneious 9.1.4 [22] and error rates estimated from scoring discrepancies between sample repeats. Parentage analyses were completed using MateSoft [25] and also inferred manually. Ploidy was inferred based on marker zygosity—homozygosity across all markers was assumed to represent a haploid hornet and any heterozygous markers deemed the sample to be diploid.

**Table 1:** Details of the microsatellite markers used in the present study, comprising details of how the markers were multiplexed and measures of gene and allelic diversity.

Marker	Multiplex reaction number	N alleles		Gene diversity		Allelic richness	
		GB	France	GB	France	GB	France
LIST2020B	1	2	6	0.354	0.566	2	5.904
R1-36	1	2	3	0.500	0.431	2	2.894
R1-80	2	2	2	0.335	0.447	2	2
R4-33	2	2	2	0.379	0.494	2	2
D2-185	3	2	3	0.316	0.493	2	2.927
R4-114	3	3	5	0.022	0.665	3	4.991
R1-169	3	1	2	0	0.378	1	2
VMA6	4	2	3	0.499	0.543	2	3
LIST2018B	4	2	5	0.397	0.631	2	4.987
D3-15	4	2	5	0.011	0.698	1.989	4.916
LIST2015	5	3	8	0.505	0.691	2.989	7.661
R4-26	5	1	5	0	0.578	1	4.81
R1-75	6	2	3	0.368	0.632	2	3
R4-100*	6	-	-	-	-	-	-
VMA8*	6	-	-	-	-	-	-
Average		2	4	0.284	0.557	1.998	3.930

* indicates marker was not used in comparisons between the French and Asian samples.

The origin of the hornets from Tetbury, Somerset and Bath was assessed by comparing the data to an existing dataset of hornet genotypes from France, South Korea and the hornet native range in South East and East Asia [24]. To account for differences in scoring between laboratories, between four and fifteen samples per multiplex were rerun from [24], allowing direct comparisons between the two datasets to be made. Two loci, VMA8 and R4-100, did not produce repeatable results and were excluded from this part of the analysis. We used the program STRUCTURE [26] to assess how the Tetbury and Somerset individuals clustered with other samples as a method of identifying their probable origin. The program was run using the admixture model and the correlated allele frequencies between populations, with a 100,000 burn-in and 200,000 MCMC iterations. The number of clusters (K) was set from 1 to 8, and 10 runs were done for each value of K. The optimum value of K was assessed using DeltaK [27], and the results per value of K aggregated and visualised using CLUMPAK [28]. The number of alleles per locus, gene diversity and allelic richness for the Great Britain (Tetbury and Somerset) samples and the French samples was calculated in FSTAT [29]; other common measures of microsatellite diversity were not calculated as the sampling for Great Britain was heavily biased by sampling multiple individuals from a single nest.

## Results

**Fig 2:**
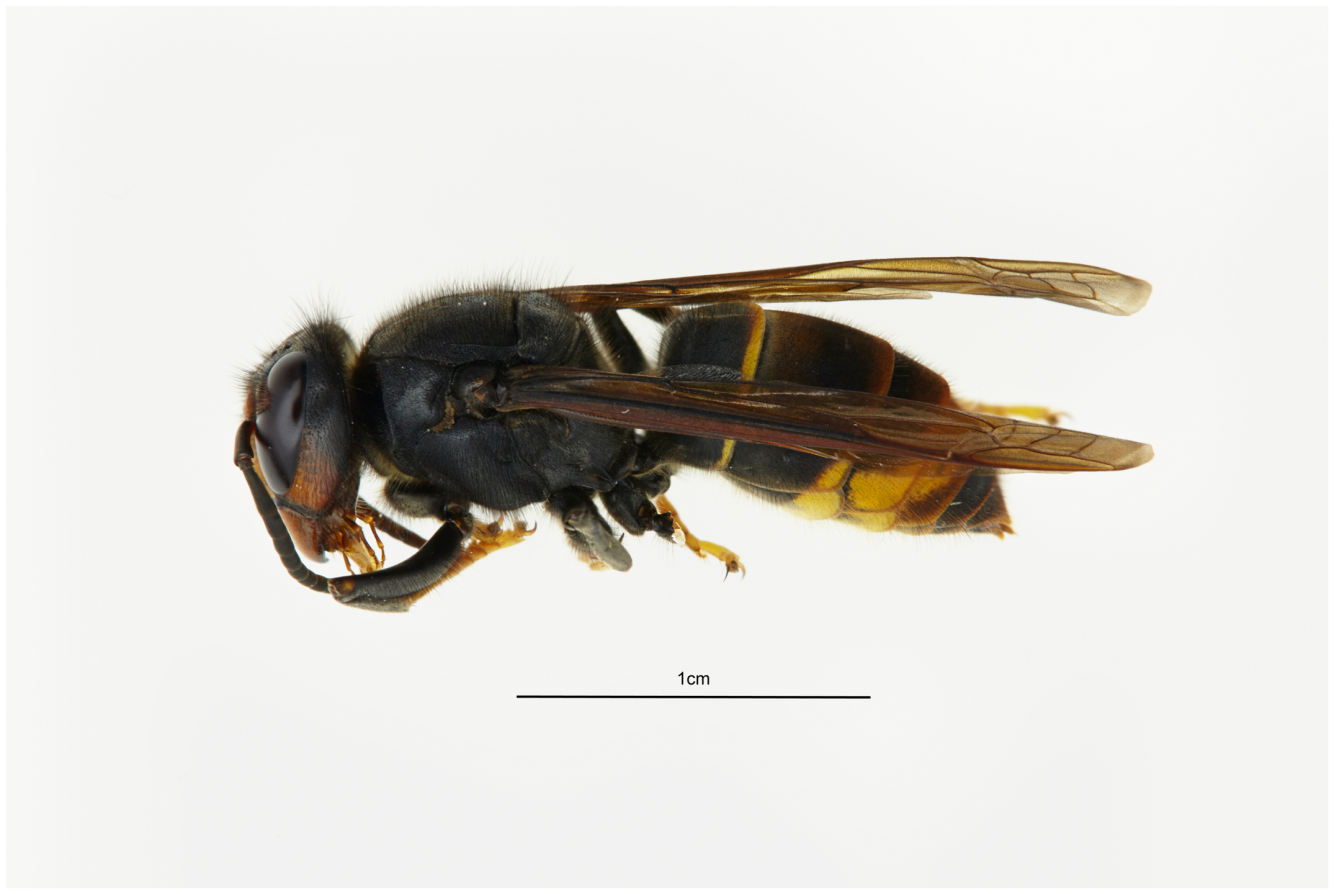
Image of *Vespa velutina*. Female *Vespa velutina* Lepeletier colour form *nigrithorax* Du Buysson captured near Tetbury, Gloucester, UK 18.IX. 2016 (‘*Tetbury1*’).

All specimens collected in Great Britain were confirmed to be *Vespa velutina* Lepeletier with the overall colouration consistent with the dark colour form *nigrithorax* Du Buysson, now found in continental Europe (Fig 2).

### Observations of foraging hornets

More than 220 field inspections of apiaries and flowering forage sites were completed in Tetbury up to a radius of 17.5 km from the eventual nest location. The majority of hornets were observed within 500 m (56 of 94 observed hornets; 60%) or 700 m (92 of 94 observed hornets; 98%) of the nest location, suggesting highly localised foraging activity. The remaining two hornets were observed foraging on ivy 1.15 km from the nest. No foraging hornets were observed in the surrounding area after the nest was destroyed, suggesting the presence of a single nest in the locality. In Somerset, no foraging hornets were observed within 20 km of the original sighting, despite 186 inspections of apiaries, forage sites and water sources, suggesting the primary nest may have failed.

### Nest observations

The Tetbury nest comprised 70 adults (57 females and 13 males), 128 teneral adults, 509 pupae, 331 pre-pupae, 584 larvae and 459 eggs. The nest contained 2,359 cells in five galleries, the largest of which had a diameter of 23 cm. The total number of hornets produced by the nest based on the diameter of the largest comb [30] was approximately 2,889. These figures are comparable with the mean number of cells (2,654) and average number of hornets per nest (3,337) produced in France in September [30]. The fresh weight of adult 19 female hornets ranged from 202–322 mg with a mean of 256 mg, whilst that of seven adult male hornets ranged from 248–326 mg with a mean of 290 mg. The dry weight of six additional individual adult females ranged from 69–112 mg. Given *V*. *velutina* queens from France weigh at least 593 mg fresh weight and 250 mg dry weight [30], these data suggest that all individual females sampled from the nest were probably workers.

### Cytochrome Oxidase I sequencing

**Fig 3:**
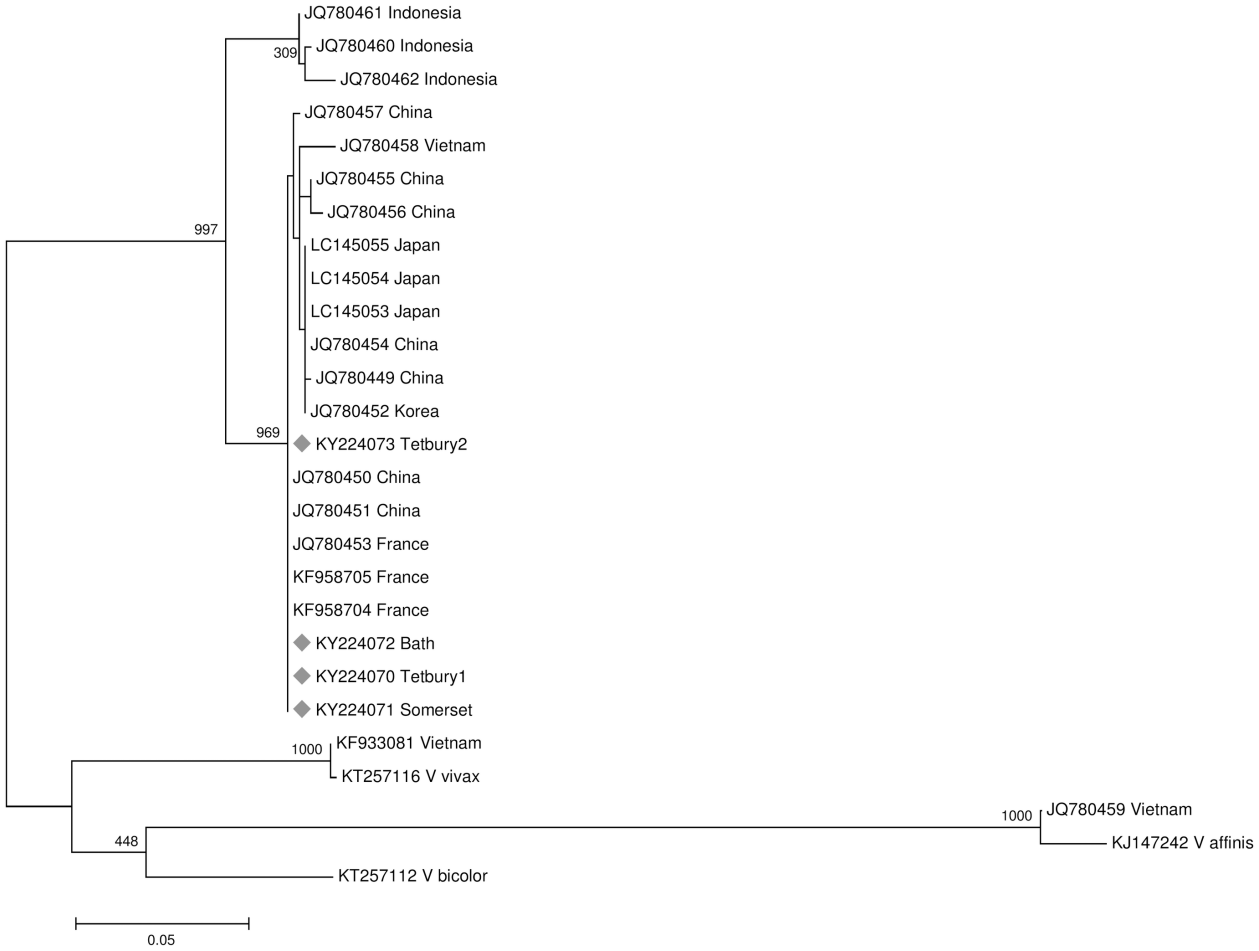
Phylogenetic tree. A PhyML Maximum-Likelihood phylogenetic tree of *Vespa velutina* COI sequences from GenBank^®^ aligned with genetic sequence data from four yellow-legged hornet specimens intercepted in Great Britain (accession numbers KY224070-KY224073, marked with diamonds). The tree is rooted with a *Vespa bicolor*, *V*. *vivax* and a *V*. *affinis* sequence (accession numbers KT257112, KT257116 and KJ147242).

COI sequences were submitted to GenBank (Accession numbers KY224070-KY224073). All hornet samples from Great Britain shared a single haplotype, which had a 96–99% sequence identity to 13 *V*. *velutina* sequences on GenBank, followed by sequences from *V*. *bicolor*, *V*. *analis* and *V*. *vivax*, all with 90% or lower sequence identity, confirming the samples were yellow-legged hornets. Two *V*. *velutina* sequences in GenBank (KF933081 and JQ780459) had less than 90% sequence identity to other *V*. *velutina* sequences, indicating that they may be misidentifications (or introgression events), which have strong sequence similarity to *V*. *vivax* and *V*. *affinis* respectively (Fig 3). The model of nucleotide substitution selected by PhyML SMS tool was GTR +G +F. In total, 20 GenBank *V*. *velutina* COI sequences were used to create a phylogenetic tree in PhyML (Fig 3). The haplotype found in Great Britain was also found in all the French *V*. *velutina* samples sequenced to date, and in samples from Jiangsu and Zhejiang in China.

### Microsatellite marker analysis

In total, 104 individual hornets were genotyped from Great Britain (S1 Table). In order to estimate the associated error rate, 12% of samples were repeated and any mismatches in genotyping recorded. The genotyping error rate was below 3% (errors were allele drop-out).

**Fig 4:**
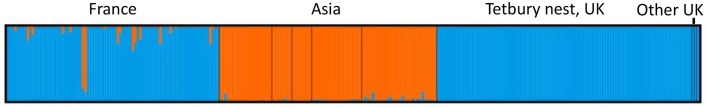
STRUCTURE plot. STRUCTURE plotwith the optimum value of K as two. All the individuals from Tetbury, Somerset and Bath formed a single cluster with samples from France (Blue), and the second cluster was formed the Asian samples (Orange). Each column represents an individual sample blocked by geographic location as indicated. France n = 85 [24]; Asia populations are divided into region from left to right Indonesia n = 21, S Korea n = 8, Vietnam n = 8, Yunnan, China n = 20 and Jiangsu & Zhejiang, China n = 30 [24]; Tetbury nest (n = 101); Other UK includes from left to right *Tetbury2* (n = 1), *Somerset* (n = 1); and *Bath* (n = 1).

The microsatellite genotypes of yellow-legged hornets from Great Britain were compared to an existing dataset comprising individuals from French and Asian populations [24]. New genotyping data were produced for between four and fifteen samples per multiplex reaction to facilitate a comparison between studies. The overall frequency of alleles in yellow-legged hornets from Great Britain was significantly higher when compared to the French samples than the Asian samples (Two-tailed paired T-test for Tetbury P<0.001; Somerset P<0.001; and Bath P<0.001). The STRUCTURE analysis suggested the optimum value of K was two, as evaluated using Delta K [27], in which all the individuals from Tetbury, Somerset and Bath formed a single cluster with samples from France, and the Asian samples formed the second cluster (Fig 4). The number of alleles per locus, gene diversity and allelic diversity for the Great Britain (Tetbury nest and Somerset) samples and the French samples are reported in Table 1. All alleles found in Great Britain were also found in France, and the number of alleles, allelic and gene diversity recorded in *V*. *velutina* samples from Great Britain was consistently lower than in samples from France.

All yellow-legged hornets determined to be male based on morphological examination (N = 19; emerged and teneral adults) were heterozygous at between 5 and 9 loci, showing they were diploid and not true haploid males. The only specimens homozygous across all 15 loci were eggs, suggesting the nest had not yet produced true adult males.

The genotypes of the male and female offspring from the Tetbury nest were used to manually infer the parental queen and drone genotypes, which were most consistent with the nest having been formed by a single queen and a single drone. The sample *Tetbury2* had a genotype consistent with being derived from the Tetbury nest, while the *Somerset* sample had alleles not found in the Tetbury nest and therefore is highly unlikely to be an offspring from that nest. Similarly, the *Bath* sample had alleles not found in either the Tetbury nest or the *Somerset* sample, and is likely to have been arrived directly from France.

## Discussion

Our COI haplotype and microsatellite profile data of *V*. *velutina* from Great Britain are consistent with the invasion representing a continued expansion of the European population. Sequences from the current study shared the same COI haplotype as has been found in all French samples sequenced to date, and in samples from Jiangsu and Zhejiang in China, the suggested origin of the French *V*. *velutina* invasion. The STRUCTURE analysis of the microsatellite data clusters the samples from Great Britain with those from France, strongly suggesting that the incursion derives from the French population. The UK samples had very low levels of gene and allele diversity compared to the French population. A formal Non-Native Species Risk Assessment for *V*. *velutina* [17] concluded that the most likely pathway by which this species could reach Great Britain would be by flight from continental Europe across the English Channel. However, the discovery of yellow-legged hornets in Tetbury and Somerset, a considerable distance from the first possible areas of landfall from continental Europe, points to human mediated movements rather than natural spread. Our observations highlight two varied pathways of entry i.e. the accidental introduction with goods such as used camping equipment brought back to the UK from Europe (‘*Bath*’) and traded timber stacks (‘*Tetbury2’*). The diversity of such pathways highlights the difficulty with intercepting yellow-legged hornets and taking early preventative action.

Microsatellite markers were a useful tool to investigate the relatedness of the hornets found during an outbreak scenario. They allowed us to trace individuals (‘*Tetbury2*’) back to the existing destroyed nest and to exclude the possibility of additional, undiscovered nests or independent incursions from France. Microsatellite marker data showed that the individual hornet caught in Somerset was not the offspring of the Tetbury nest, providing useful data to inform spread models and direct future surveillance. The microsatellite data also suggested that the Tetbury nest was likely formed by a single queen that had mated with a single drone. As queens in France typically mate with between 2.4–4.1 drones [24], this suggests that the Tetbury queen may have mated in an area of low drone density, perhaps from the advancing invasion front in continental Europe or from within Great Britain. In France, data suggest that the proportion of nests reported is low compared to the total number of nests present [31], and while nest destruction has slowed the invasion, local control has a limited impact on the spread of *V*. *velutina* once the hornet has established within a region [32]. Our data indicate that the founding queen from the Tetbury nest and Somerset hornet either represent two independent introductions of live hornets into the UK from a genetically related area of low nest density, or are outbreaks from an earlier common incursion. The relatively small distance between the Tetbury nest and Somerset hornet (54 km) is noteworthy given an invasion speed of up to 82 km/year [31]. Taken together, our data highlight that future monitoring should concentrate in the Tetbury and Somerset areas in case other *V*. *velutina* nests remain undetected.

Microsatellite data also indicated that all the anatomical males from Tetbury were diploid and hence not ‘true’ males. This is consistent with observations in France where over 97% of early males were diploid [33]. *Vespa velutina* has a complementary sex determination system, which is common within the order Hymenoptera [33]. Inbreeding increases the chance of homozygosity at the sex locus, resulting in diploid males that are thought to be sterile [24], although a triploid *V*. *velutina* male has been reported [33]. Persistent observations of diploid males may suggest that *V*. *velutina* have short mating flights [34]. *Vespa velutina* colonies showing ‘normal’ development produce males about 15 days before the emergence of the first reproductive females [30]. True haploid males had been laid in the Tetbury nest, but were only present at the egg stage. Coupled with the absence of any females large enough to be considered queens, it seems likely that the nest was destroyed before the production of sexual adult females. Although it is worth noting that different environmental conditions can lead to body size changes in some hymenoptera species [35], and therefore body mass may not rule out the presence of founder queens when considered in isolation.

While multiple nests of *V*. *velutina* have been found within 1 km of apiaries containing foraging hornets, the foraging range of the nest remains unknown [18]. Our data indicate that *V*. *velutina* demonstrates highly localised foraging i.e. within 700 m of the nest, a behaviour that will likely contribute to underreporting of incursions and make the successful eradication of this pest more challenging. The pollinator population faces many threats, including land use change, lack of forage, the use of pesticides and the introduction of non-native pests and diseases [36, 37]. The yellow-legged hornet represents another significant pressure on pollinators in Great Britain, and whilst challenging to achieve, affecting a successful eradication policy will clearly benefit pollinator health.

## Supporting information

S1 Table Sample metadata. Details of the samples of yellow-legged hornets from Great Britain used in this study. The life stage of the individuals is given in column B, the sex of the individuals (as determined by morphological traits) is given in column C and the individuals presumed to be homozygotes (i.e. are homozygous for al markers tested) are indicated in column E. Microsatellite marker data for all the samples are given in columns F to AI.
(XLSX)
